# Hybrid Plasma–Liquid Functionalisation for the Enhanced Stability of CNT Nanofluids for Application in Solar Energy Conversion

**DOI:** 10.3390/nano12152705

**Published:** 2022-08-06

**Authors:** Ruairi J. McGlynn, Hussein S. Moghaieb, Paul Brunet, Supriya Chakrabarti, Paul Maguire, Davide Mariotti

**Affiliations:** Nanotechnology and Integrated Bio-Engineering Centre (NIBEC), Ulster University, Newtownabbey BT37 0QB, UK

**Keywords:** solar–thermal, plasma functionalisation, carbon nanotubes

## Abstract

Macroscopic ribbon-like assemblies of carbon nanotubes (CNTs) are functionalised using a simple direct-current-based plasma–liquid system, with oxygen and nitrogen functional groups being added. These modifications have been shown to reduce the contact angle of the ribbons, with the greatest reduction being from 84° to 35°. The ability to improve the wettability of the CNTs is of paramount importance for producing nanofluids, with relevance for a number of applications. Here, in particular, we investigate the efficacy of these samples as nanofluid additives for solar–thermal harvesting. Surface treatments by plasma-induced non-equilibrium electrochemistry are shown to enhance the stability of the nanofluids, allowing for full redispersion under simulated operating conditions. Furthermore, the enhanced dispersibility results in both a larger absorption coefficient and an improved thermal profile under solar simulation.

## 1. Introduction

Zero-carbon renewable energy sources such as solar energy provide an alternative method to satisfy the continued increase in energy demand without the concomitant environmental degradation caused by burning fossil fuels. One method to obtain thermal energy from solar sources is to utilise direct-absorption solar collectors (DASCs) [1], where the working fluid acts both to absorb incident solar radiation and to transfer the captured energy to a hot-water tank or other storage systems. As typical heat-transfer fluids (e.g., water, ethylene glycol, oil) are poor absorbers in the visible-light region [1,2,3], it is necessary to introduce additives to effectively capture a significant portion of this high-energy region. Early additives included micron-scale carbonaceous particles, which whilst increasing the absorption of light in the visible region, also had the negative effects of fouling the transparent covers and clogging the pumping systems, and therefore limited the lifetime and efficiency of the overall system [1,2]. Reduction in system fouling has been achieved by adding nanoscale particles to produce nanofluids, whilst also providing enhanced optical absorption with lower particle concentrations, as demonstrated for aluminium [4]. Beyond this, other nanoparticles have been tested, including gold [5,6], silver [7,8], and copper oxide nanoparticles [9,10,11], providing the opportunity to tune the optical absorption of the working fluid by blending a carefully selected range of these additives.

By comparison to these noble- or transition-metal additives, carbon-based materials are relatively Earth-abundant and eco-friendly. Additionally, some of these materials—such as graphene/graphene oxide [12,13] and carbon nanotubes (CNTs) [14,15,16,17,18]—demonstrate extremely high optical absorption, approaching blackbody levels [19], which when paired with exceptional thermal conductivity [20,21] present attractive options for nanofluid additives. CNTs are potentially one of the most attractive options, as they undergo a phenomenon called “shear thinning”, which is beneficial to their application in DASCs. Where the fluid is constantly pumped, the CNTs can align in the direction of the liquid flow, which at low CNT concentrations can result in a working fluid with a viscosity even lower than that of the base fluid alone [22]. At greater shear rates, entangled and agglomerated CNTs can be deagglomerated, enhancing the stability of the nanofluid [23].

Initial studies have demonstrated the high suitability of functionalised CNTs as nanofluid additives, with much of the thermal conductivity and optical properties of the CNTs lent to the nanofluid even at low CNT contents. At 150 parts per million carboxylate-functionalised CNTs, Karami et al. obtained an extinction coefficient of 4.1 cm^−1^ and a 32.2% thermal conductivity increase when using water as the base fluid [15]. Furthermore, the authors noted that higher dispersions were possible, although these absorbed too much light to perform any optical measurements. Hordy et al. [18] addressed the suitability of water- and glycol-based nanofluids for usage at 85 °C and 170 °C, respectively, with oxygen-grafted plasma-functionalised CNTs. Beyond this, they also confirmed a storage stability of over eight months in glycol-based fluids, losing only 8.6% of the effective concentration. It was noted that the water-based fluid lost approximately 5% of the effective concentration per month. This is echoed in other similar works, where even ultralow contents of CNT additives result in near-perfect absorption (0.2 volume percent) and substantially improved thermal conductivity over the base fluid alone (0.508 W m^−1^K^−1^) [24]. Studies of CNTs within DASC devices have also yielded promising results, with carboxyl-functionalised CNTs in a 30:70 ethylene glycol:water mixture demonstrating an improvement in collector efficiency of between 10 and 29% compared to the base fluid alone, with increasing CNT contents and flow rates giving greater enhancements [14]. The improvement was attributed to the 54.4% increase in thermal conductivity at 100 ppm CNTs coupled with an extinction coefficient of over 5 cm^−1^. In a tubular collector configuration, similar benefits of CNT additives were found [25], with a 0.01 weight percentage of surfactant-stabilised CNTs yielding a thermal efficiency of 80%, which was 37.9% greater than that of a comparable opaque receiver plate. The full-scale system was operated for 45 days and 15 h per day, with no degradation to the pumping system noted, although there was evidence of some deposition of materials near the pump impeller.

CNTs are inherently hydrophobic and, thus, require modification of the surface chemistry to enable superior dispersion and stability within a fluid matrix [26], as is the case with each of the above examples. Two main methods exist to remedy this: coating with surfactants, and chemical treatments. Surfactants can provide a repulsive force, due to zeta potential, by creating a surface charge, which results in improved stability and lifetime of the nanomaterials in solution [27]. Whilst these have been demonstrated to provide high stability, there are three main limiting factors in the use of surfactants for stabilisation: (1) the surfactant can degrade at temperatures above 100 °C [15,16,27,28,29], although some are capable of working up to 300 °C, such as sodium dodecylbenzenesulfonate [16]; (2) the coated surfactant can lower the thermal conductivity of the individual carbon additives, though in some cases this may be overcome by the enhanced distribution of the absorbing additives in the fluid [28,29]; and (3) the surfactants can modify the refractive index and scattering of the particles, inducing a spectral shift [30]. As an alternative to surfactants, the surface functional groups of the CNTs can be modified by using strong oxidants such as sulfuric or nitric acids [15,16], or weak oxidants such as potassium hydroxide [15], to add hydroxyl and carboxylic groups. However, these chemical methods often require protracted treatment times and the use of harsh, often hazardous chemicals. This highlights the need for an alternative pathway to produce CNTs that are stable in aqueous solutions and at higher temperatures.

One option for this is to utilise plasma treatments, which can both modify the surface chemistry and remove excess amorphous carbons [26]. With plasma-based treatments, reduced reaction times and the avoidance of aggressive chemicals—such as concentrated acids—are key improvements over traditional covalent chemical methodologies [26]. A range of low-pressure plasma systems have been utilised to provide oxygen- and nitrogen-based functional groups, including microwave-source [31,32] and radio-frequency systems [33,34,35,36,37], with the radio-frequency systems also reporting the additional benefit of the removal of excess amorphous carbons. Eliminating the vacuum equipment provides the benefits of reducing the capital investment involved in establishing a process, as well as simplifying the processing itself by allowing direct processing of liquids and colloids, such as in plasma-induced non-equilibrium electrochemistry [38,39,40]. The use of plasma-induced non-equilibrium electrochemistry for the functionalisation of nanocellulose has been demonstrated in water-based suspensions, with both oxygen and nitrogen functional groups added depending on the plasma conditions and electrolytes [41].

In this work, an atmospheric-pressure plasma treatment is demonstrated for the formation of oxygen and nitrogen functional groups on the sidewalls of macroscopic CNT assemblies. This system utilises a direct-current (DC) source between a submerged electrode (CNT ribbon) and a microplasma counter-electrode, across an electrolyte solution with or without a nitrogen precursor [42].

## 2. Materials and Methods

### 2.1. CNT Synthesis

The CNTs in this work were produced in a floating-catalyst chemical vapour deposition system that produces ribbon-like macroscopic assemblies of continuous length [43]. Ferrocene, thiophene, and methane were flown in a furnace [43] with hydrogen as a carrier gas, and where the iron catalyst nanoparticles were formed by the thermal decomposition of ferrocene powder. After the hot zone of the furnace, the material began to cool, and a dense web of dark carbon material formed in the centre of the work tube. This aerogel was sufficiently strong that it could be drawn out with a rod to form a long black “sock”.

To produce the ribbon-like CNT assemblies, gas flows of 115.5 standard cubic centimetres per minute (sccm) of hydrogen through the ferrocene bubbler, 90 sccm of hydrogen through the thiophene bubbler, a methane flow rate of 160 sccm, and a carrier hydrogen flow rate of 1470 sccm were flown through a tube furnace maintained at 1290 °C. In the context of this work, the term “ribbon” is used to describe the material obtained by this process after pressing the macroscopic CNT aerogel between two glass microscope slides for 1 min under a 2 kg force to create a 3–4 cm long, 0.5–1.0 cm wide, and 1–10 µm thick flat ribbon-like material.

### 2.2. CNT Functionalisation

**Figure 1:**
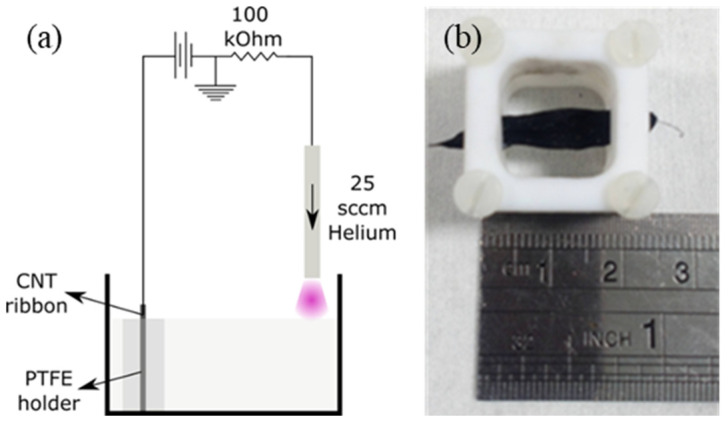
(**a**) The configuration of the plasma-induced non-equilibrium electrochemistry system used to treat the ribbons, and (**b**) a photograph illustrating the PTFE holder used to secure the carbon nanotubes.

The CNT ribbon samples were subjected to different treatments and compared to the pristine samples. Prior to plasma functionalisation, all samples discussed in this manuscript were annealed for 15 min at 350 °C in a Carbolite VMF 10/15 furnace with a standard air atmosphere. Samples not pre-treated with this annealing step are documented in the Supplementary Materials, and show a slightly lower overall performance. A plasma-induced non-equilibrium electrochemistry system was used for the functionalisation of the CNT ribbons (Figure 1). In this system, direct-current microplasma is generated in helium between a nickel capillary tube (inner diameter of 0.7 mm and outer diameter of 1 mm) and the electrolyte surface. The helium gas flow of 25 sccm (controlled by a mass flow controller, MKS Instruments, UK) ensures that the plasma is largely formed in helium gas, with a small amount of turbulent mixing with the surrounding air expected. A voltage of 1.3 kV was applied to the CNT ribbons using a Matsusada AU-10 * 15 power supply, and all samples of CNTs were treated at a constant current of 10 mA for 15 min. Over the treatment period, the applied voltage dropped from approximately 1.3 kV to approximately 1 kV in order to maintain the constant current. The CNT ribbon was secured in place by a PTFE support frame (Figure 1b), which prevented the ribbon from moving during the treatment. An exposed surface of 1.5 cm in length and between 0.5 cm and 1 cm in width was exposed to the electrolyte solution. We used two different solutions for the plasma–liquid treatment. The first comprised ethanol and water at a ratio of 1:9, with a volume of 18 mL, hereafter referred to as “Plasma–liquid”. The second, “Plasma–liquid with EDA”, included a 10% volume of ethylenediamine with the same stock solution of 1:9 ethanol:water—again with a volume of 18 mL. After the plasma treatments, the ribbons were submerged in a beaker of distilled water for 5 min.

### 2.3. CNT Ribbon Characterisation

X-ray photoelectron spectroscopy (XPS) was used in this study to analyse the elemental composition and the chemical bonding. The spectrometer was an ESCALAB XI^+^ instrument, (Thermo Fisher, East Grinstead, United Kingdom ). The base pressure during spectral acquisition was above 5 × 10^−7^ mbar, achieved using an Edwards E2M28 rotary vane pump. The main background gas in the analysis chamber was argon at all points during the loading, pumping, and measurements. The excitation source was a monochromated aluminium anode with an excitation energy of 1486.68 eV operated at approximately 15 kV and 15 mA, giving a source power of 225 W. The work function of the spectrometer was determined by the software as 4.68 eV. The recorded spectra included C 1s, O 1s, N 1s, Fe 2p, S 2p, and Pt 4f (reference), which were acquired sequentially, with a total acquisition time of 662 s. With the selected scan parameters, the energy resolution was 0.1 eV for high-resolution spectra and 1 eV for survey spectra. The size of the analysed sample area was 650 µm, taking the form of an elongated circle. The samples were stored overnight in standard lab conditions before loading into the spectrometer. The transfer procedure within the fast-entry lock of the spectrometer included exposure to 2 × 10^−6^ mbar, achieved with an Edwards RV5 rotary vane pump (Edwards, West Sussex, United Kingdom) in less than 10 min prior to XPS analysis. Charge compensation, by means of an electron beam, was applied via a flood gun operated at 100 µA. The charge referencing method was performed by shifting the asymmetric Pt 4f_7/2_ peak of freshly sputter-cleaned Pt foil to 71.2 eV. The foil was pressed onto the surface of the CNT ribbons with a copper clip. The sputter-cleaning was performed with a monoatomic argon ion gun held at 4000 eV and 15 mA at an angle of 30° to the sample over a 1.5 mm wide square. This was expected to give a sputter rate of 0.97 nm s^−1^ for tantalum(V) oxide.

A Horiba LabRAM 300 spectrometer with a 632.81 nm helium–neon fixed-wavelength laser was used to obtain Raman spectra. Measurements were taken 1 cm apart for a total of five measurements per sample to assess the variance of the graphitisation within the sample. An 8th-order polynomial baseline subtraction was performed using the system software (LabSpec, Horiba). The D band was considered the peak at 1335 cm^−1^, with the G band at 1580 cm^−1^. The third peak at 1622 cm^−1^ was considered as the D’ band, and was fitted to exclude it from the G-band area. The area enclosed by each of these components was calculated by integrating the peaks with OriginPro 9 software, where a peak was defined as having a minimum height of 15% of the data and a width between 5% and 15% of the data. Finally, the area ratio of G:D was calculated by dividing the two integrated areas.

The effect of the treatments on the hydrophobicity of the CNT ribbons was assessed by contact angle measurements with a KSV Instruments CAM 200 system. A drop of distilled water was placed on the surface of the ribbons and allowed to settle for 15 s before an image was taken. The contact angle was then calculated by the system software and recorded. This was performed 5 times per sample to generate an average and a standard deviation.

### 2.4. Nanofluid Preparation and Optical Characterisation

The nanofluids were prepared by weighing the CNT ribbons on a microbalance and then adding the ribbon and the necessary volume of ethylene glycol to a sample vial to produce a concentration of 25 mg L^−1^. The CNT ribbon was then dispersed as separate CNTs using a Sonics VCX 130PB system with a 3 mm probe diameter, operated at 70% power for 300 s, giving an average tip power of 10 W over the duration.

The optical properties of the produced nanofluids were tested with a PerkinElmer Lambda 650 system with a 150 mm integrating sphere. This system allows for the determination of the constituent parts of light attenuation by the sample: transmittance, scattering, and absorption. Furthermore, the absorption, scattering, and extinction coefficients can be calculated. A quartz cuvette with a path length of 1 cm was filled with pure ethylene glycol and used as a reference. Measurements were carried out both in standard transmittance mode and within the integrating sphere. The calculations for the absorption, scattering coefficients, and power are provided in the Supplementary Materials.

For UV–Vis data collected from day 120 onwards, the instrument was upgraded to a PerkinElmer Lambda 1050+, although the sample integrating sphere unit was migrated to the new system.

### 2.5. Methods for the Assessment of the Solar–Thermal Conversion

**Figure 2:**
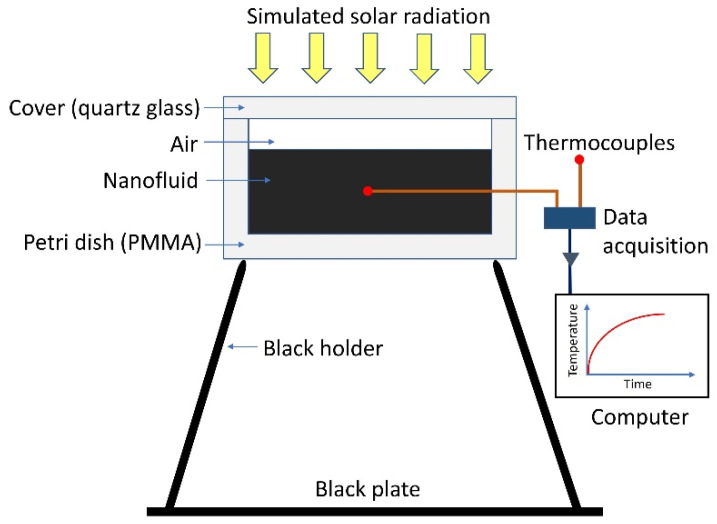
Schematic of the experimental solar–thermal conversion setup used to measure the time-dependent temperature variation of nanofluids exposed to simulated solar irradiation.

Solar simulation experiments were performed with a 150 W xenon arc lamp with a 25 mm diameter uniform beam (LS0106, Lot-QuantumDesign). This lamp was calibrated to an intensity of one sun (1000 W m^−2^) across the wavelength range of 400–1100 nm. As illustrated in Figure 2, a small-volume transparent vessel (>92% transmittance in visible light [44]) was produced from poly(methyl methacrylate), with the following dimensions: internal diameter of 21 mm, base thickness of 1.7 mm, and sidewall thickness of 2.5 mm. A cover made from a 1.8 mm thick quartz disc of 21 mm in diameter was used as a lid for the vessel to minimise heat loss by natural convection during the solar–thermal conversion (STC) experiments. The transmittance of the cover was experimentally measured and found to be ≈94% (see Supplementary Materials) over a wavelength range of 300–2500 nm, i.e., a net intensity of 940 W m^−2^ reached the nanofluid surface. Then, 800 µL of each nanofluid was added, giving a fluid height of approximately 2.3 mm from the base of the vessel. This small volume allowed for a more uniform heat distribution within the sample. T-type thermocouples were used to measure the sample and ambient air temperatures, with the data recorded onto a PC via a TC-08 thermocouple data acquisition module (Omega). A black plate was positioned 3 cm below the sample vessel to capture transmitted light and prevent reflection back into the sample. No thermal insulation was used in these experiments, in order to allow for more accurate calculation of the various parameters. This setup allowed for temperature measurements of static-state nanofluid samples exposed to solar irradiation. On this basis, we were able to then calculate the total power absorbed, which was the sum of the stored thermal power within the nanofluid and the heat loss to the surroundings. Full details of the calculations are provided in the Supplementary Materials.

## 3. Results

### 3.1. CNT Ribbon Chemical Analysis

**Figure 3:**
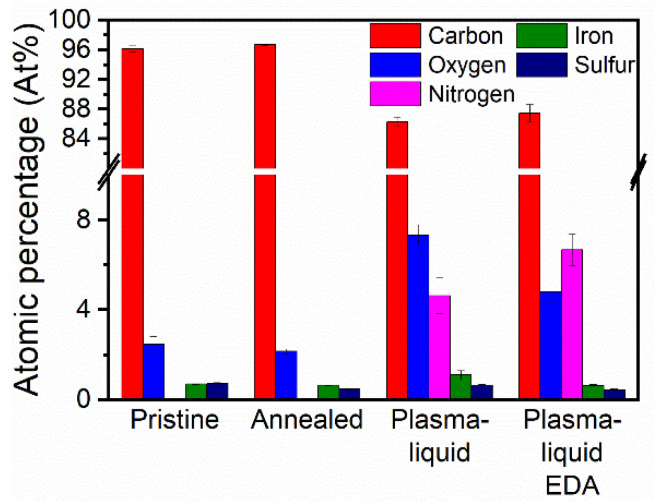
X-ray photoelectron spectroscopy summary of atomic percentages by element for oxygen and nitrogen. The remaining percentage is composed of carbon.

XPS was used to assess the functionalisation of the CNTs by the plasma–liquid treatments, with a summary of the atomic percentages given in Figure 3. The oxygen content was 2.5 at% for the pristine sample, and was slightly decreased after the annealing step to 2.2 at%. Both the pristine and annealed samples showed no nitrogen. When CNTs were subjected to either plasma treatment, an increase in oxygen content was observed, and the presence of nitrogen was then detected. The oxygen content increased to 7.4 at% with the plasma–liquid treatment, with a lesser increase to 4.8 at% with the plasma–liquid with EDA treatment. The nitrogen content was 5 at% for the plasma–liquid sample and 7 at% when EDA was also included. Whilst nitrogen was not seen in the pristine or annealed-only materials, the plasma treatment in both electrolyte solutions resulted in the inclusion of nitrogen within the sample. Reactive nitrogen species such as nitrate, nitrite, and the corresponding nitric acid can be generated in the plasma due to the interaction of the plasma and the surrounding atmospheric nitrogen. These reactive species can then be dissolved within the liquid, where they may interact with the CNTs if they are sufficiently long-lived [45]. As a result, even in the absence of a deliberately dissolved nitrogen precursor, the plasma–liquid samples presented a relatively high content of nitrogen. Sulphur and iron were present due to the catalysts, and were within the range 0.4–0.7 at% and 0.6–1.1 at%, respectively, for all samples.

**Figure 4:**
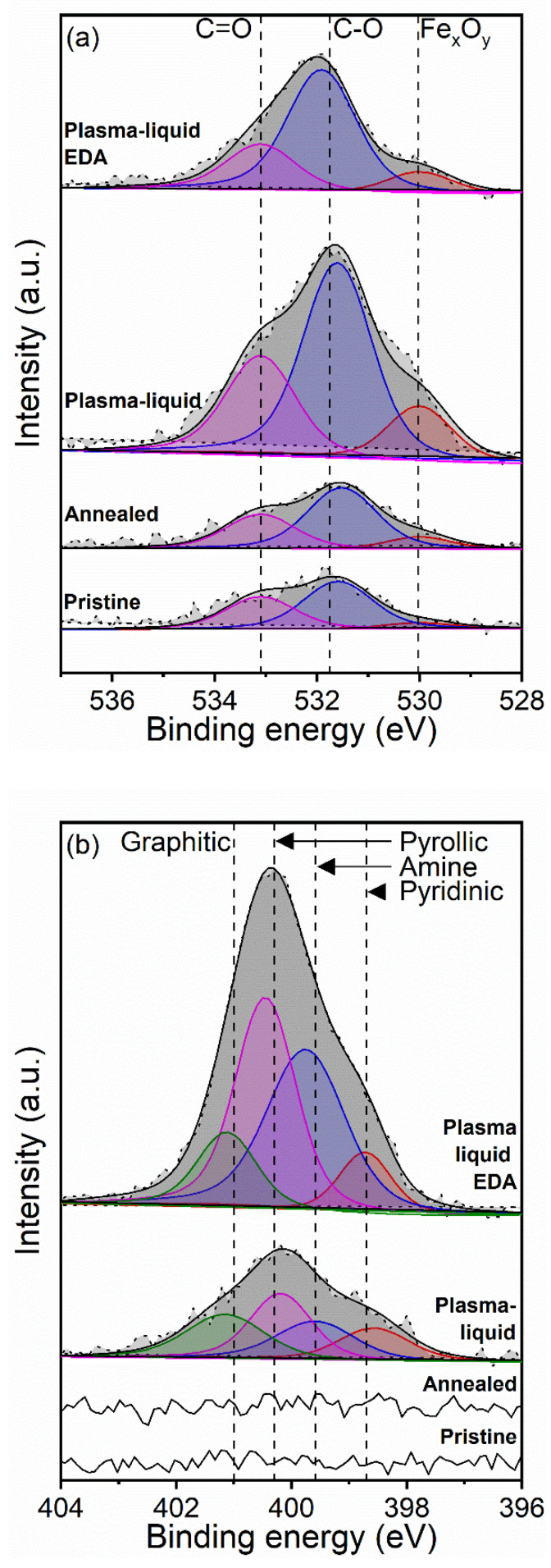
High-resolution X-ray photoelectron spectra of (**a**) oxygen and (**b**) nitrogen for pristine and pre-annealed carbon nanotube ribbons.

Figure 4 shows the high-resolution spectra for oxygen and nitrogen. All of the O 1s spectra present two features corresponding to the carbon–oxygen bonding: C–O and C=O. Annealing results in an increase in the C=O peak area (by 6%) and an increase in the C–O peak area (by 29%) compared to the pristine sample. Both peak areas are much larger for both the plasma–liquid-treated samples. In particular, the plasma–liquid sample exhibits the most oxygen functionalisation, with the largest peak areas for C=O and C–O species—increased by 204% and 311%, respectively, with respect to the pristine sample. Whilst the plasma–liquid with EDA treatment also enhances the C=O peak area (61% increase) and C–O peak area (383% increase) components significantly compared to the pristine material, its total carbon–oxygen bonding is lower than that of the plasma–liquid-treated material without EDA. The peak that is linked to Fe*_x_*O*_y_* develops after annealing, and is further enhanced after either plasma treatment. This could be due to the removal of some of the surface carbons by the plasma treatment, which exposes more catalysts to the XPS measurements. It is also possible that the catalysts are subject to a degree of oxidation during the different treatments. However, we should note that there was some variability in the catalyst density in the sampled areas; for instance, the plasma–liquid sample appeared to have a higher atomic concentration of iron (Figure 3), which would justify the higher contribution to the Fe_x_O_y_ component in the O 1s signal in Figure 4. Figure 4b includes the N 1s spectra for all four samples; however, the annealed and pristine samples clearly indicate that no nitrogen was detected in these cases. However both of the plasma treatments resulted in the inclusion and development of nitrogen bonds with features including pyridines, amines, pyrroles, and graphitic nitrogen groups [46,47,48,49,50,51]. Whilst the graphitic nitrogen contents and pyridinic contents were comparable for the plasma–liquid and plasma–liquid with EDA samples, the peak areas of the pyrollic and amine components were vastly different. The pyrollic peak area was shown to increase from 3812 CPSeV to 12,099 CPSeV, and the amine peak area increased from 2879 CPSeV to 12,020 CPSeV when EDA was included in the electrolyte solution. This demonstrates that pyrollic and amine bonding are directly impacted by adding EDA to the electrolyte.

**Figure 5:**
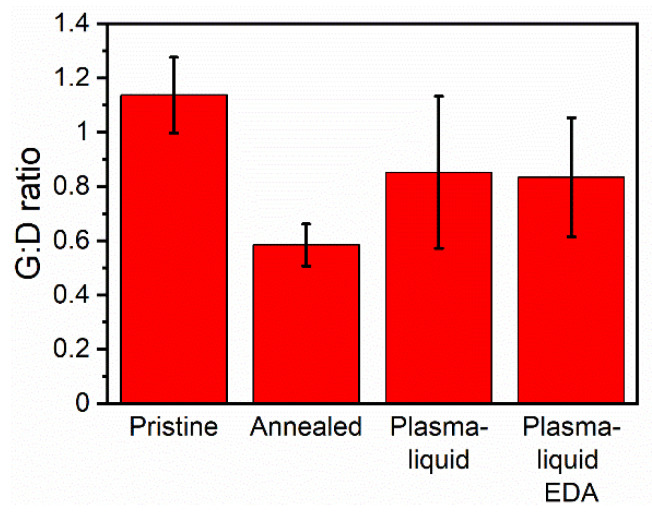
Summary of the G-band to D-band area ratios determined by Raman spectroscopy.

Raman spectroscopy was employed to assess the G-band to D-band area ratio (G:D ratio), which gives an indication of the graphitisation of the CNTs and whether the treatment results in defects (reduction in G:D). We should note that the synthesis method of the CNT ribbons introduces an intrinsic variability of the G:D ratio, which has an average value of 1.3 with a 0.2 standard deviation (determined over five spots per sample for 108 samples). Figure 5 shows a reduction in the G:D ratio of the annealed samples from 1.1 to 0.6. The G:D area ratio recovered after either plasma–liquid treatment, to approximately 0.8. This can be explained by the inclusion of graphitic nitrogen in the carbon lattice, which could reduce the number of missing atoms in the graphitic lattice and, therefore, give a strong lower D-band peak area.

**Figure 6:**
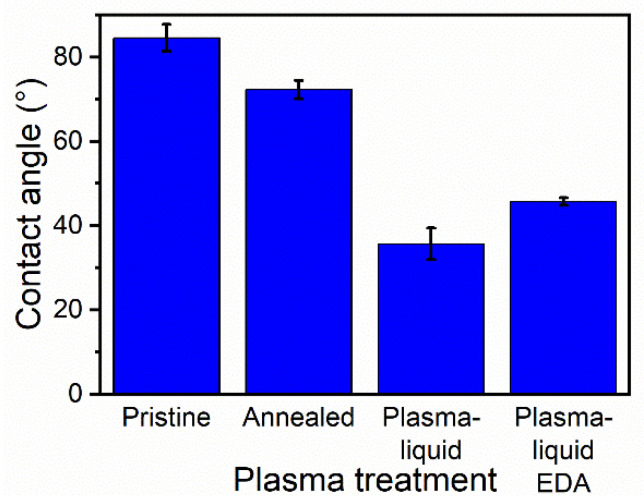
Summary of the contact angle measurement results for the 6 different carbon nanotube treatments.

The change in the hydrophobicity of the CNT ribbons after each plasma treatment was assessed using contact angle measurements (Figure 6). The untreated sample showed a contact angle of 84°, with a large drop to 46° for the samples treated with plasma–liquid in the presence of EDA. A remarkable decrease to 35° was measured for the plasma–liquid-treated samples in the base electrolyte of ethanol and water only. This represents a substantial shift towards a hydrophilic regime, and is suggested to be due to the functionalisation of the plasma–liquid treatment, as the intermediary annealing step results in a contact angle of only 72°.

### 3.2. Nanofluid Optical Characterisation and Solar–Thermal Conversion

**Figure 7:**
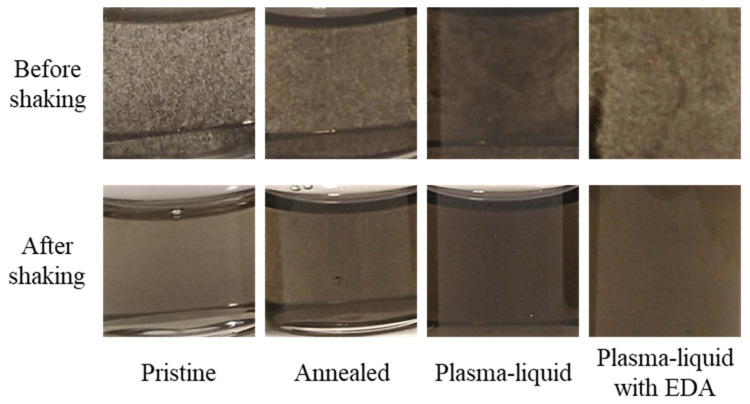
Photographs of the nanofluids after 809 days of storage before shaking, and after 2 vigorous shakes by hand. Note that the photographs have been modified to enhance the contrast and brightness to highlight flocculation.

The sedimentation and redispersion of the CNTs in nanofluids were monitored over a period of more than 2 years (809 days exactly). The nanofluids were allowed to settle in a dark cupboard with no disturbances (Figure 7). The pristine sample showed clear signs of flocculation. The same, to a lesser degree, was observed in the annealed sample and the plasma–liquid EDA sample. The plasma–liquid sample was the one that appeared to be the least impacted by long-term storage. All four vials were then shaken with no more than two vigorous shakes by hand (Figure 7). Shaking improved the dispersion of all samples, although clear differences remained, with the plasma–liquid sample still visually presenting the best dispersion, as it did before shaking. This was consistent with the contact angle measurements, which showed the best hydrophilicity for the CNTs treated with the plasma–liquid sample. More results at different timing and for non-annealed samples are documented in the Supplementary Materials.

**Figure 8:**
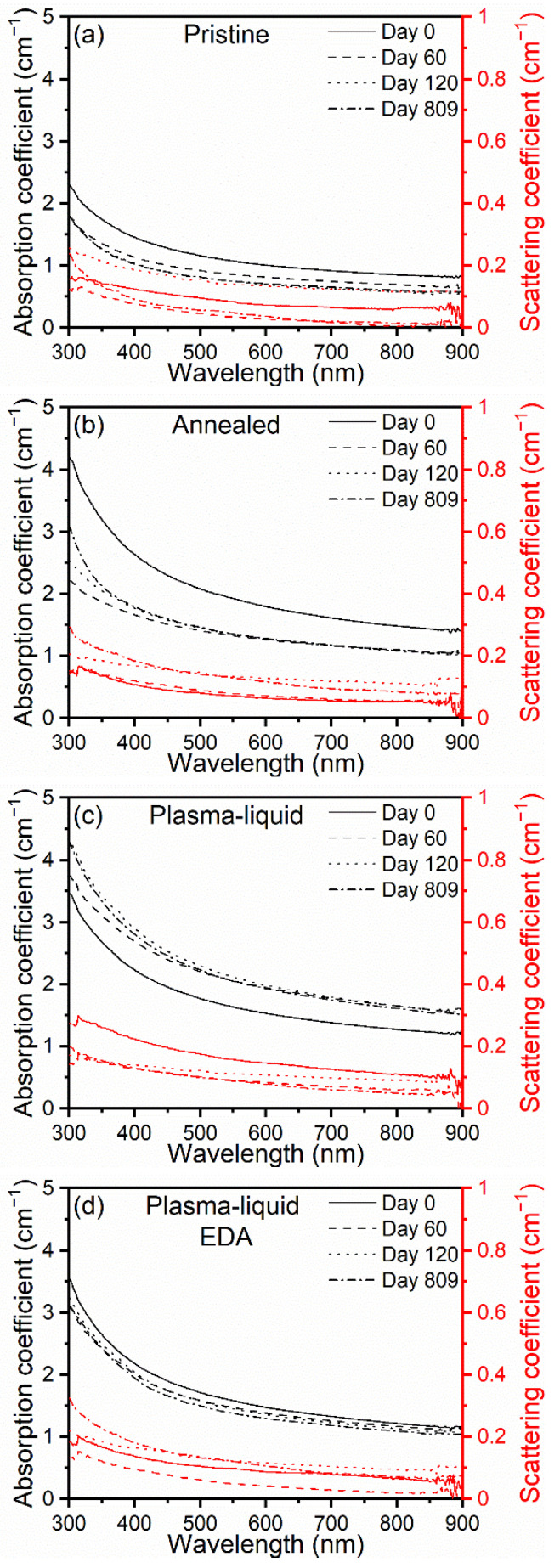

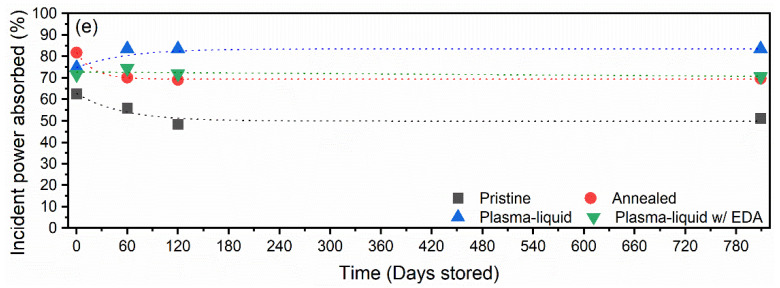
The values of absorption and scattering coefficients obtained from ultraviolet–visible spectroscopy via the transmittance port and an integrating sphere (**a**–**d**). The percentage of incident power absorbed for each carbon-nanotube-based nanofluid over 120 days (**e**).

These observations are useful to understand the mechanisms of sedimentation, but they are a qualitative assessment and are not fully representative of real-life applications, where the nanofluids are subject to circulation, with continued opportunities for mixing and gaining the advantages of shear-thinning mechanisms [22,23]. Hence, before UV–Vis spectroscopy, each sample was shaken vigorously by hand twice to redisperse the CNTs. UV–Vis spectroscopy measurements of the CNT nanofluids showed that all samples exhibited the same optical features and, therefore, that the treatment type did not appear to affect the qualitative nature of the light’s interaction with the nanofluids (Figure 8). The nanofluid samples presented strong attenuation across the scanned range and a Pi-plasmon absorbance in the UV region at approximately 250–260 nm [52,53]. However, this region was also dominated by the absorption of the ethylene glycol base fluid; as such, it was not assessed here. The differences between the samples lie in the intensity of the optical coefficients and the stability over time, and it should be noted that the scattering coefficients in each case are an order of magnitude smaller than the absorption coefficients. Over the time period assessed, the treated samples possessed higher absorption coefficients across the full spectral range when compared to the pristine and annealed samples. The absorption coefficients of both of the plasma-treated samples showed very little change over time, while the pristine and annealed samples exhibited clear reductions in the absorption coefficients over the ~2-year period. The annealed sample, however, was slightly better in terms of both absorptivity and stability when compared to the pristine sample. Similarly, the plasma–liquid sample showed better absorption and stability when compared to the plasma–liquid EDA sample. For the plasma–liquid sample, we observed an increase in the absorption coefficient across the spectral range, and we attributed this to the progressive disentanglement of the CNTs, which might have improved over time and after shaking at day 60. This was corroborated by a decrease in the scattering coefficient over the same period, suggesting an improved dispersion. These results are consistent with the trends observed in the contact angle measurements (Figure 6) and photos (Figure 7). The scattering coefficients are somewhat more difficult to interpret, as their values, which are much lower than the absorption coefficients, are more susceptible to experimental variations, and such differences could be considered negligible. This is particularly the case when comparing the scattering curves over time for the same samples. In general, we can confirm that the scattering coefficient is higher for samples with a higher absorption coefficient, confirming that flocculation and sedimentation are at least in part responsible for the different optical properties of the four samples.

The absorption coefficient measured for each nanofluid at different times was then used to calculate the percentage of power absorbed from a spectrum of solar light at AM1.5G.ach and with a nanofluid, for a theoretical fluid depth of 1 cm (see the Supplementary Materials and Equation (16)). On day 0, the pristine sample absorbed 63% of the solar power, whilst the plasma–liquid with EDA and the plasma–liquid samples absorbed 72% and 75%, respectively. The annealed sample also exhibited a very high percentage of absorbed power (82%). After 60 days, a substantial decrease in the absorptive properties of the pristine and annealed samples was found, dropping to 56% and 70%, respectively. Consistent with the measured absorption coefficients, the absorbed power increased to 84% and 74% for the plasma–liquid and plasma–liquid EDA samples, respectively. By day 120, the power absorbed by the pristine sample continued to fall, plummeting to 48%. After more than 2 years of storage, it could be seen that the plasma–liquid and plasma–liquid EDA samples retained their absorptive properties, with a calculated power absorbed above 80% and above 70%, respectively. The pristine CNT nanofluid remained below 50%, and the annealed samples remained below 70%. These results strongly reinforce that the “plasma–liquid” treatment can produce nanofluids with superior optical properties and enhanced stability.

These observations clearly show that both plasma-treated samples gained in terms of absorbed power compared to the pristine sample, likely due to the added functional groups. Hence, these not only play a role in the stability of the CNTs, but also can partially improve light absorption. The difference in stability was also obvious, as both plasma-treated samples were very little affected after more than 2 years. We also observed that the plasma–liquid sample outperformed the plasma–liquid with EDA sample. This could also be linked to differences in nitrogen- and oxygen-based functional groups. This suggests that oxygen-based groups appear to be more effective in promoting light absorption and, therefore, should be preferred for enhancing the overall absorption.

**Figure 9:**
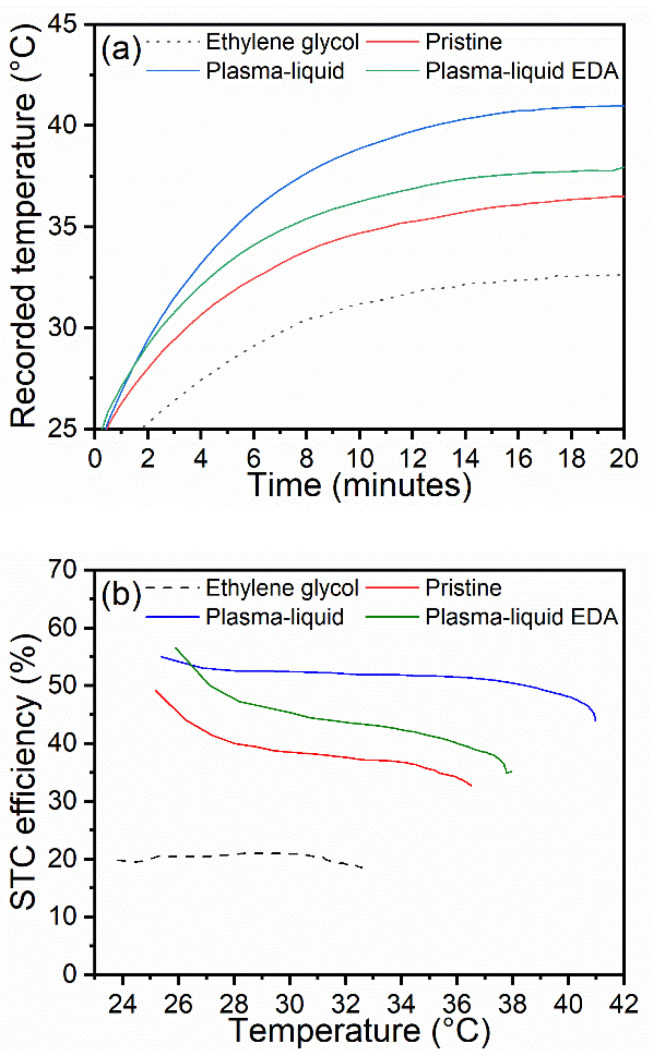
(**a**) Variation in temperature of the nanofluids and ethylene glycol over 20 min of exposure to simulated solar radiation, and (**b**) their resulting solar–thermal conversion efficiency after accounting for heat lost.

Solar–thermal conversion efficiency was assessed for each of the treated CNT types; this was carried out on day 60 (Figure 9). In terms of the recorded temperatures of the nanofluids (Figure 9a), the pristine CNT sample showed the lowest temperature rise, with an increase of 12.3 °C over 20 min. Improving upon this, the plasma–liquid with EDA sample demonstrated a temperature increase of 13.1 °C. As expected from the previous UV–Vis measurements and absorbed power calculations, the plasma–liquid sample demonstrated the greatest temperature rise under the solar simulator, with a temperature increase of 16.7 °C. For each CNT additive, the performance of the nanofluid was enhanced over pure ethylene glycol, which only provided a 9.6 °C temperature increase. These results align with the incident power absorbed, as shown in Figure 8e. Figure 9b shows a substantial enhancement in the STC efficiency—in particular with the “Plasma–liquid” CNT nanofluid, where an efficiency of approximately 50% was achieved. In addition to the greatest achieved STC efficiency, the “Plasma–liquid” sample also retained this over the greatest temperature range, surpassing 40 °C before dipping. By comparison, the “Pristine” and “Plasma–liquid with EDA” samples showed a decrease in the STC efficiency at much lower temperatures, even below 30 °C. The ethylene glycol did not demonstrate a decrease in efficiency with temperature, although this can be ascribed to the substantially lower maximum temperature recorded during the solar–thermal experiments.

## 4. Conclusions

We used plasma-induced non-equilibrium electrochemistry to functionalise the surface of CNT ribbons and enhance their dispersion within ethylene glycol for application in solar-to-thermal energy conversion. In particular, the nanofluids with CNTs that showed a combined nitrogen-based and high oxygen-based functionalisation—the “plasma–liquid” samples—performed exceptionally well. These showed a reduction in the contact angle from 84° to 35°, along with corresponding improvements in hydrophilicity and dispersion, leading to the enhancement of the absorption coefficient, reaching values greater than 2 cm^−1^ at 600 nm, with much greater values above 5 cm^−1^ observed at the logarithmic Van Hove peak at 256 nm. Solar simulations showed that this “plasma–liquid” additive results in the greatest temperature increase—to above 40 °C—as well as a solar–thermal conversion efficiency of 50%. The long-term performance of the treated nanofluids was assessed, with values of 80% incident radiation absorption retained after 67 months of storage, with only a brief manual shake required to fully redisperse the material prior to UV–Vis measurements, highlighting the readiness for industrial application.

## Data Availability

All relevant data can be made available upon request to the corresponding author.

## Supplementary Materials

The following supporting information can be downloaded at: https://www.mdpi.com/article/10.3390/nano12152705/s1, Figure S1: UV–Vis transmittance measurement of the fused quartz cover used to seal the Petri dish for solar–thermal experiments; Figure S2: Schematic diagram showing (a) the heat loss mechanism from the nanofluid to the ambient surroundings, and (b) the thermal circuit of the thermal resistances during heat loss; Figure S3: Scanning electron micrographs of the different carbon nanotube ribbons after sonication and drop-casting onto a silicon wafer; Figure S4: Photographs of the non-annealed nanofluids after 809 days of storage (a) before shaking and (b) after 2 vigorous shakes by hand; Figure S5: Scanning electron micrographs of the different carbon nanotube ribbons after sonication and drop-cast onto a silicon wafer; Figure S6: X-ray photoelectron spectroscopy summary of atomic percentages by element for oxygen and nitrogen; Figure S7: High-resolution X-ray photoelectron spectra of oxygen and nitrogen for pristine and pre-annealed carbon nanotube ribbons; Figure S8: Raw spectral data of the carbon nanotubes prior to dispersion; Figure S9: Summary of the G-band to D-band area ratios determined by Raman spectroscopy; Figure S10: Summary of the contact angle measurement results for the 6 different carbon nanotube treatments; Figure S11: Photographs highlighting the short-term flocculation effect on stored nanofluids without disturbance; Figure S12: The values of absorption and scattering coefficients obtained from ultraviolet–visible spectroscopy via the transmittance port and an integrating sphere. The percentage of incident power absorbed for each carbon-nanotube-based nanofluid over 120 days is presented in the fourth chart; Figure S13: (a) Variation in temperature of the nanofluids and ethylene glycol over 20 min of exposure to simulated solar radiation, with small improvements noted for the plasma-treated and annealed samples. (b) The resultant solar thermal conversion efficiency after accounting for heat lost; Figure S14: Photographs taken during the solar–thermal conversion experiments; (a) shows the “pristine” sample, whereas (b) is a photograph of the “plasma–liquid” conditions; Table S1: A table of symbols and constants used in the solar simulation calculations. References [3,14,54,55,56,57,58,59,60,61] are cited in the Supplementary Materials.
