# Supplementary material for: Hybrid Plasma–Liquid Functionalisation for the Enhanced Stability of CNT Nanofluids for Application in Solar Energy Conversion
Source: Nanomaterials (Basel). 2022 Aug 6;12(15):2705. doi: 10.3390/nano12152705 (PMC9370158; doi:10.3390/nano12152705)
Supplement: Supplementary file 1 [file nanomaterials-12-02705-s001.zip › nanomaterials-1825131-supplementary-1.pdf]

Supporting information

Hybrid Plasma–liquid functionalisation approach for the enhanced stability a of CNT nanofluids for application in solar energy conversion

Ruairi J. McGlynn *, Hussein S. Moghaieb, Paul Brunet, Supriya Chakrabarti, Paul Maguire and Davide Mariotti *

Nanotechnology and Integrated Bio-Engineering Centre (NIBEC), Ulster University, Newtownabbey BT37 0QB, United Kingdom

*Corresponding authors: d.mariotti@ulster.ac.uk and r.mcglynn@ulster.ac.uk

S1. Calculations for the absorption and scattering coefficients

All calculations in this section are wavelength dependent. By using the standard transmittance port, a value for the transmitted light can be obtained. This transmittance measurement is simply the fraction of light which is still collimated when leaving a sample of path length, *l*, divided by the total incident light on the sample for the same light path;

$$T = \frac{I(l)}{I_0} \quad (S1)$$

Through the use of an integrating sphere it is possible to evaluate the proportion of light affected by the cumulative effect of transmittance (*T*) and scattering (*S*). The percentage of light absorbed (absorptance, Abs) is considered to be the percentage of light which is not transmitted or scattered:

$$Abs = 1 - (T + S)_{meas} \quad (S2)$$

From the basic measurements, a number of calculations need to be carried out to extract relevant physical parameters such as extinction coefficient, μ, absorption coefficient, μ_a and scattering coefficient, μ_s. The main assumption is that in the spectral range of interest, the base fluid, use as reference, has very high transmittance, i.e. negligible absorptance and scattering (*Abs*, *S* << *T*). In our case this is largely true for the range 450–850 nm. Here these calculations and derivations are described.

By subtracting the signal of the measurement in the transmittance port, it is possible to isolate the percentage of signal due to scattering:

$$S = (T + S)_{meas} - T \quad (S3)$$

The light transmitted through a length of fluid, *l*, will have undergone a number of extinction events determined by the extinction coefficient:

$$I(l) = I_0 e^{-\mu l} \quad (S4)$$

Using Equation S1 and taking the natural logarithm a function for the extinction coefficient can be derived:

$$ln\, T = -\mu l \quad (S5)$$

$$\mu = -\frac{ln\, T}{l} \quad (S6)$$

The total incident light of a given wavelength absorbed by a fluid of depth, x, can be expressed as the integral between 0 and l of light intensity at x multiplied by the absorption coefficient.

$$I_A(l) = \int_0^l I(x)\mu_a dx \quad (S7)$$

$$I_A(l) = \int_0^l I_0 e^{-\mu x}\mu_a dx \quad (S8)$$

$$I_A(l) = \mu_a I_0 \int_0^l e^{-\mu x} dx \quad (S9)$$

$$I_A(l) = \mu_a I_0 \frac{(1-e^{-\mu l})}{\mu} \quad (S10)$$

The percentage absorption has been defined as the absorbed light intensity at a given fluid depth divided by the incident light intensity, Equation S12. By substituting this into

Equation S11 and taking logarithms gives an equation for the absorption coefficient, Equation S15.

$$\frac{I_A(l)}{I_0} = Abs \tag{11}$$

$$Abs = \frac{I_A(l)}{I_0} = \mu_a \frac{(1-e^{-\mu l})}{\mu} \tag{S12}$$

$$\mu_a = \frac{\mu}{(1-e^{-\mu l})}(Abs) \tag{S13}$$

$$\mu_a = -\frac{ln\, T}{l(1-T)}(Abs) \tag{S14}$$

The scattering coefficient is simply calculated by:

$$\mu_s = \mu - \mu_a \tag{S15}$$

By using the American Society for Testing and Materials (ASTM) reference spectra [1] as the incident irradiance (W m^{-2}) for light at each wavelength, the power per unit area that is absorbed by the nanofluids for a given pathlength has been calculated. Replacing I_0 with the power per m^2 for a given wavelength, *Q*, from the reference spectra, Equation S11 can be rewritten as:

$$Q_A(l) = \int \mu_a Q_0 \frac{(1-e^{-\mu l})}{\mu} \tag{S16}$$

Finally, by integrating the power absorbed across all measured wavelengths the power absorbed by a nanofluid for a range of different path lengths can be calculated.

S2. Solar thermal conversion experimental procedures of and related calculations

A table of symbols and constants is provided in Table S1 to aid in the understanding of the calculations below.

Table S1 A table of symbols and constants used in the solar simulation calculations.

Symbol	Definition	Value
ΔT_{nf}	Change in nanofluid temperature	
A_s	Area of the irradiated surface	
$A_{s,i}$	Surface area of the inner wall	
$A_{s,o}$	Surface area of the outer wall	
A_{wall}	Surface area of the wall	
$c_{p,bf}$	Thermal conductivity of base fluid	
$c_{p,nf}$	Nanofluid specific heat capacity	
$c_{p,np}$	Thermal conductivity of nanomaterial	
$h_{comb,bot}$	Combined heat transfer coefficient (free convection + radiation) of the ambient air at the adjacent surface of the petri dish bottom	5 $Wm^{-2}k^{-1}$
$h_{comb,top}$	Combined heat transfer coefficient (free convection + radiation) of the ambient air at the adjacent surface of the cover	8 $Wm^{-2}k^{-1}$
$h_{comb,wall}$	Combined heat transfer coefficient (free convection + radiation) of the ambient air at the adjacent surface of the petri dish wall	10 $Wm^{-2}k^{-1}$
H_{wall}	Height of the petri dish wall	
I_o	Incident radiation	
k_{air}	Thermal conductivity of air	0.0242 $Wm^{-1}k^{-1}$
k_{bf}	Thermal conductivity of base fluid	
k_{cov}	Thermal conductivity of the cover	1.4 $Wm^{-1}k^{-1}$
k_{np}	Thermal conductivity of nanomaterial	
k_{pd}	Thermal conductivity of petri dish	0.2 $Wm^{-1}k^{-1}$

m_{nf}	Nanofluid mass	
q_g	Heat generated	
R_{bot}	Total resistance from nanofluid to the ambient through the axial downward direction	
$R_{cd,air}$	Conduction resistance of the still air layer	
$R_{cd,bot}$	Conduction resistance of the petri dish bottom	
$R_{cd,cov}$	Conduction resistance of the cover	
$R_{cd,wall}$	Conduction resistance of the petri dish wall	
$R_{comb,bot}$	Combined (convection + radiation) thermal resistance to heat transfer from the outer surface of the petri dish bottom to the ambient air and surrounding	
$R_{comb,top}$	Combined (convection + radiation) thermal resistance to heat transfer from the outer surface of the cover to the ambient air and surrounding	
$R_{comb,wall}$	Combined (convection + radiation) thermal resistance to heat transfer from the outer surface of the petri dish wall to the ambient air and surrounding	
r_i	Inner radius of the petri dish	
r_i	Outer radius of the petri dish	
R_{top}	Total resistance from nanofluid to the ambient through the axial upward direction	
R_{wall}	Total resistance from nanofluid to the ambient through the radial direction	
t_{air}	Thickness of the still air layer	
t_{bot}	Thickness of the petri dish bottom	
t_{cov}	Thickness of the cover	
t_{ird}	Irradiation time	
T_{nf}	Recorded nanofluid temperature at any irradiation time during the STC process	
$T_{nf,i}$	Nanofluid initial temperature	
v_{nf}	Nanofluid volume	
V_{nf}	the volumes of the nanofluid	
V_{np}	the volumes of the nanomaterial	
$\eta_{sth_{exp}}$	Experimental solar-thermal conversion efficiency	
ρ_{bf}	Density of base fluid	
ρ_{nf}	Nanofluid density	
ρ_{np}	Density of nanomaterial	
φ	Volume fraction of the nanomaterial dispersed in the base fluid	

Each nanofluid was loaded into the petri dish and covered with the quartz glass disc, which was measured by UV–Vis to transmit approximately 94 % of light between 300nm and 2500 nm as seen in Figure S1.

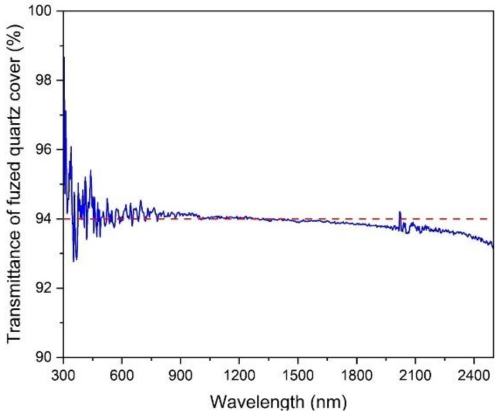

Figure S1. UV–Vis transmittance measurement of the fused quartz cover used to seal the petri dich for solar thermal experiments.

The collector with the nanofluid was then mounted on the plastic holder and coaxially positioned below the simulator output at a vertical working distance of 3 cm. The temperature was monitored until thermal equilibrium with the room temperature was reached. Once thermal equilibrium was reached, the solar simulator was turned on at 1000 W/m^2 radiation intensity, while recording the change in the nanofluid temperature until the end of irradiation time. An energy balance was used for calculating the experimental STC efficiency ($\eta_{sth_{exp}}$) of each nanofluid, as well as the base fluid. The incident radiation, I_o, per unit area of the irradiated surface, A_s, is attenuated by absorption and scattering within the nanofluid volume, resulting in heat generation, q_g, from the STC process. Therefore, the nanofluid STC efficiency, which is the ratio between the heat generated and the incident radiation intensity per unit surface area, can be obtained with

$$\eta_{sth} = \frac{q_g}{I_o A_s} \quad (S17)$$

The rate of heat generated within the nanofluid, q_g, can be determined from the change in the nanofluid temperature with respect to its initial temperature during the irradiation time taken for this change at a given mass and heat capacity and taking into account the heat loss to ambient air and surroundings.

$$q_g = m_{nf} c_{p,nf} \frac{\Delta T_{nf}}{t_{ird}} + q_l = \rho_{nf} V_{nf} c_{p,nf} \left(\frac{T_{nf} - T_{nf_i}}{t_{ird}} \right) + q_l \quad (S18)$$

The density and specific heat capacity of the nanofluid, which is a mixture of the nanomaterial and the base fluid, can be determined from the following correlations at a given volume fraction.[2,3]

$$\varphi = \frac{V_{np}}{V_{nf}} \quad (S19)$$

$$\rho_{nf} = \varphi \rho_{np} + (1 - \varphi) \rho_{bf} \quad (S20)$$

$$c_{p,nf} = \frac{\varphi\left(\rho_{np} c_{p,np}\right) + (1 - \varphi)\left(\rho_{bf} c_{p,bf}\right)}{\rho_{nf}} \quad (S21)$$

$$\mu_{nf} = \mu_{bf}(1 + 2.5\varphi) \quad (S22)$$

$$k_{nf} = k_{bf} \left(\frac{k_{np} + 2k_{bf} + 2\varphi\left(k_{np} - k_{bf}\right)}{k_{np} + 2k_{bf} - 2\varphi\left(k_{np} - k_{bf}\right)} \right) \quad (S23)$$

Heat lost from the nanofluid is driven by the increase in the nanofluid temperature above the ambient and surrounding temperatures as a result of the heat generated from the STC process. This heat loss is due to the heat flux from the nanofluid through the petri dish and the cover walls by conduction and then to the ambient air and surrounding walls by natural convection and radiation, respectively. Using the thermal-circuit method, see Figure S2, the

total heat loss can be calculated from the ratio of the temperature difference between the nanofluid and ambient air to the total thermal resistance between them.
The heat loss is distributed in parallel through three directions: radially through the wall, axially upward through the still air layer and cover, and axially downward through the petri dish bottom. This heat loss by natural convection and radiation can be combined using the so-called combined heat transfer coefficient and substituting in Newton's law of convection.[4] Similar studies assumed the free convection coefficient of ambient air to be constant.[5–8] However, this value depends on the fluid properties and the characteristics of the surface exposed to that fluid. Therefore, the natural convection coefficient has a different value for each surface (top, wall and bottom) resulting in different heat loss rates in the three directions.[4] Based on that, the free convection coefficient was calculated using valid empirical correlations of external natural heat transfer by convection for hot-upward horizontal plate (cover top surface), hot-downward horizontal plate (petri dish bottom surface) and vertical wall (petri dish and cover lateral walls), which are detailed in reference,[4] and these values were found to be 8 $Wm^{-2}K^{-1}$, 5 $Wm^{-2}K^{-1}$ and 10 $Wm^{-2}K^{-1}$, respectively.

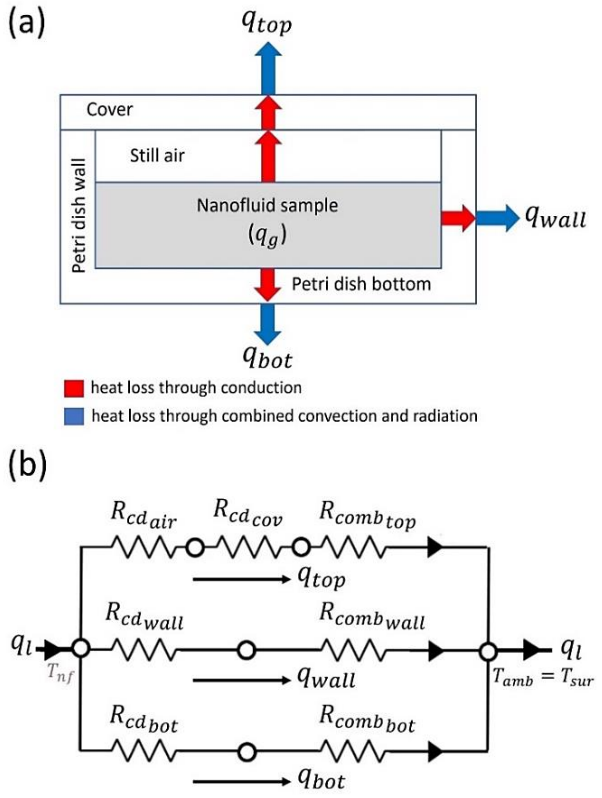

Figure S2. Schematic diagram shows (**a**) heat loss mechanism from the nanofluid to ambient and surroundings and (**b**) thermal circuit of the thermal resistances during heat loss.

$$q_l = q_{wall} + q_{top} + q_{bot} = \frac{\Delta T}{R_{th}} \quad (S24)$$

$$R_{th} = \left(\frac{1}{R_{wall}} + \frac{1}{R_{top}} + \frac{1}{R_{bot}}\right)^{-1} \quad (S25)$$

$$R_{wall} = R_{cd_{wall}} + R_{comb_{wall}} \quad (S26)$$

$$R_{top} = R_{cd_{air}} + R_{cd_{cov}} + R_{comb_{top}} \quad (S27)$$

$$R_{bot} = R_{cd_{bot}} + R_{comb_{bot}} \quad (S28)$$

$$R_{cd_{wall}} = \frac{ln(r_o/r_i)}{2\pi k_{pd} H_{wall}} \quad (S29)$$

$$R_{cd_{top}} = \frac{t_{cov}}{k_{cov}A_{s,o}} \tag{S30}$$

$$R_{cd_{bot}} = \frac{t_{bot}}{k_{pd}A_{s,o}} \tag{S31}$$

$$R_{cd_{air}} = \frac{t_{air}}{k_{air}A_{s,i}} \tag{S32}$$

$$R_{comb_{wall}} = \left(h_{comb_{wall}} * A_{wall}\right)^{-1} \tag{S33}$$

$$R_{comb_{top}} = \left(h_{comb_{top}} * A_{s,o}\right)^{-1} \tag{S34}$$

$$R_{comb_{bot}} = \left(h_{comb_{bot}} * A_{s,o}\right)^{-1} \tag{S35}$$

S3. Scanning electron microscopy of dispersed carbon nanotube nanofluid

Scanning electron microscopy (SEM) is carried out using a Schottky field emission Hitachi SU5000 instrument with an X-Max^{N} 80 silicon drift detector from Oxford Instruments. Silicon wafers are broken into small sections and sonicated in IPA to clean the surface. The CNT nanofluids were dropcast onto cleaned wafer sections, with the excess fluid subsequently evaporated on a hot plate at 80 °C. Double-sided copper tape was used to both attach and ground the sample to the SEM sample stub for measurements.

SEM measurements were taken to assess any morphological changes induced by the treatment and sonication of the CNTs, Figure S3. Significant overlap and entanglement of individual CNTs can be seen in all samples, with the level of entanglement appearing to be independent of the treatment. Furthermore, catalyst particles from the ribbon synthesis process is also clearly visible in all the samples due to the larger atomic number of the particles compared to the carbon, allowing for more secondary electrons to be generated.[9,10] Ultimately, the plasma-liquid treatment does not appear to have any noticeable effect on the morphology.

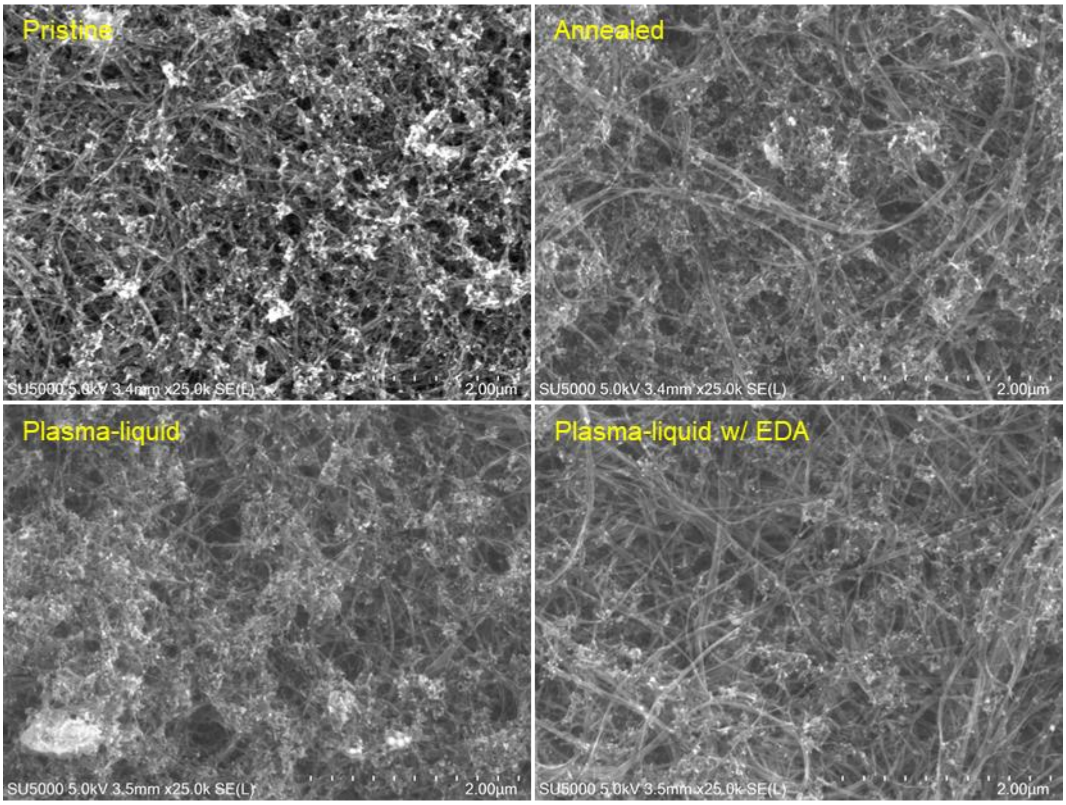

Figure S3. Scanning electron micrographs of the different carbon nanotube ribbons after sonication and drop-cast onto a silicon wafer.

S4. Non-annealed samples: characterisation and optical performance

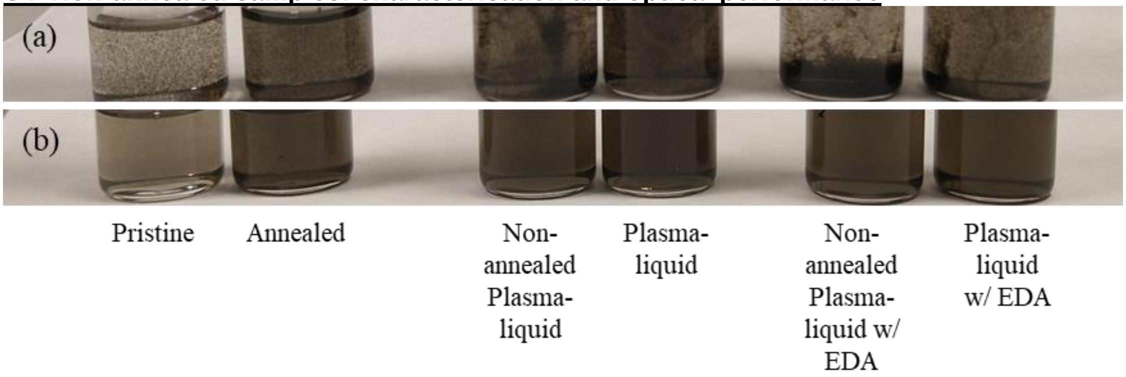

Figure S4 Photographs of the non-annealed nanofluids after 809 days of storage (**a**) before shaking and (**b**) after 2 vigorous shakes by hand.

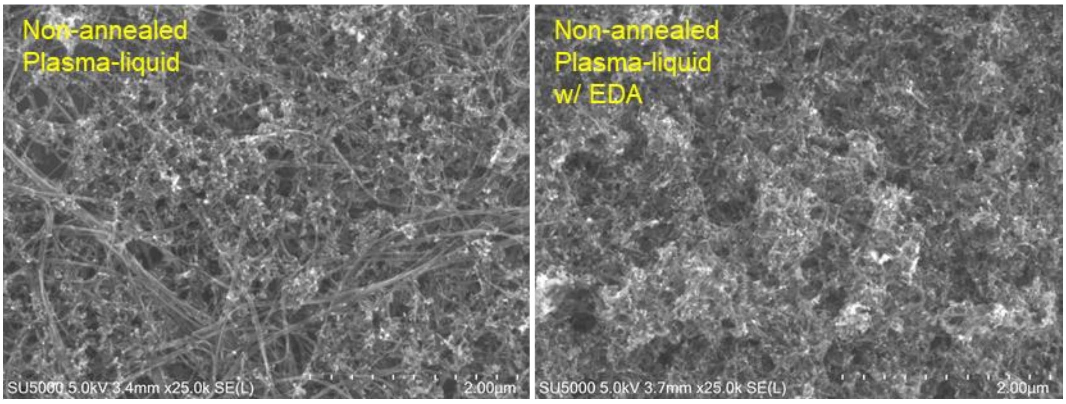

Figure S5. Scanning electron micrographs of the different carbon nanotube ribbons after sonication and drop-cast onto a silicon wafer.

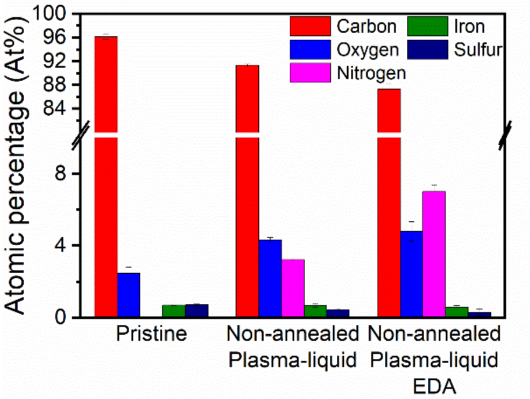

Figure S6. X-ray photoelectron spectroscopy summary of atomic percentages by element for oxygen and nitrogen. The remaining percentage is comprised of carbon. Whilst the values of the oxygen and nitrogen content are comparable with and without annealing for the plasma-liquid with EDA, both the oxygen and nitrogen content are lower for the plasma-liquid samples without the prior annealing step.

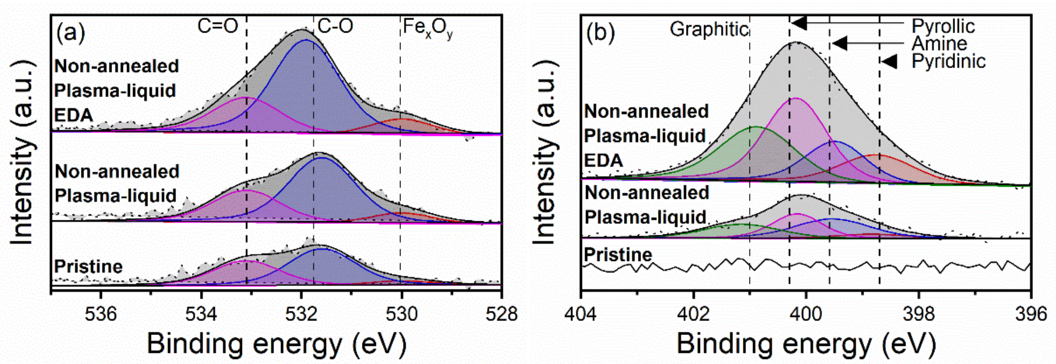

Figure S7. High resolution x-ray photoelectron spectra of (**a**) oxygen and (**b**) nitrogen for pristine and pre-annealed carbon nanotube ribbons.

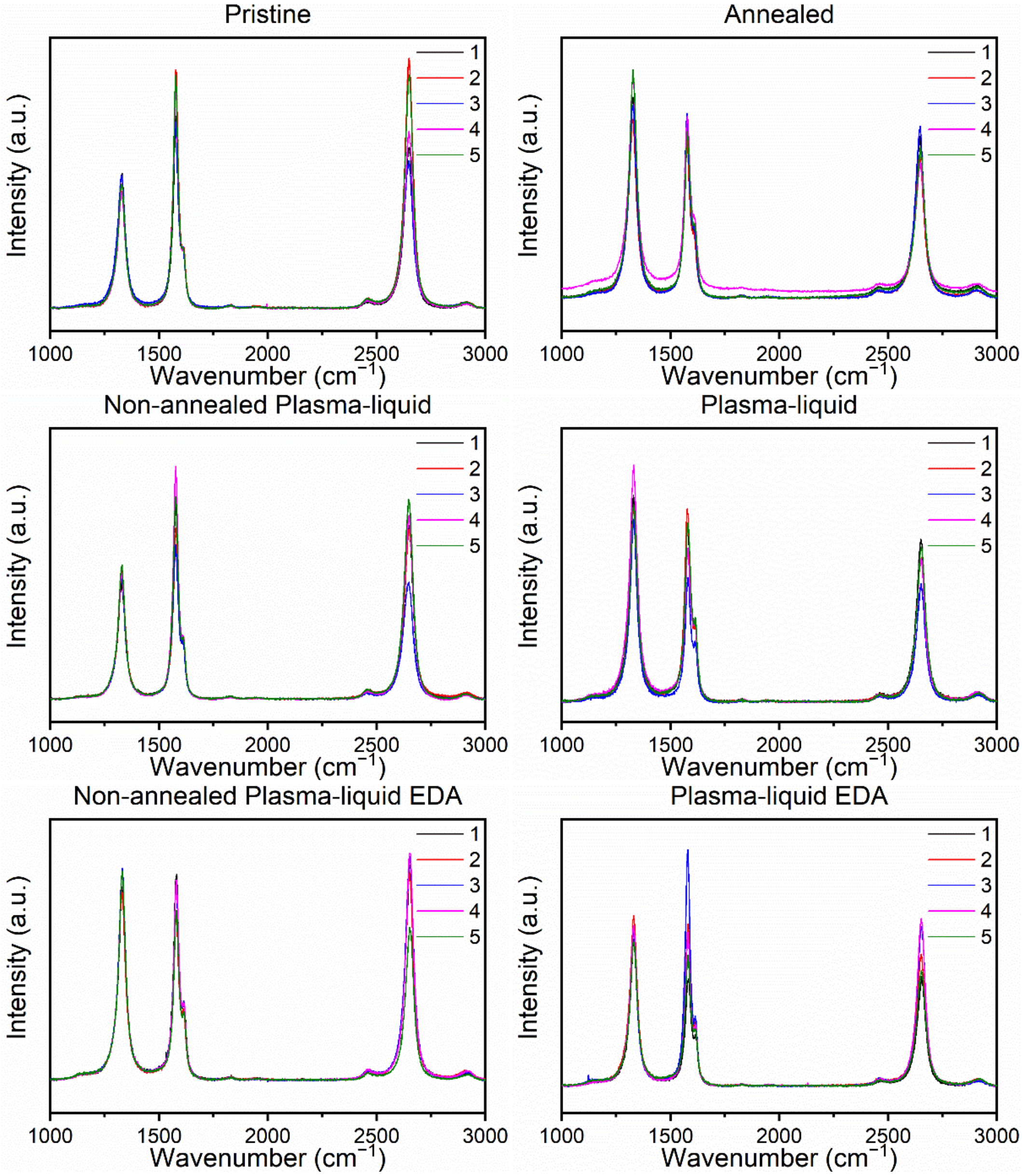

Figure S8. Raw spectral data of the carbon nanotubes prior to dispersion.

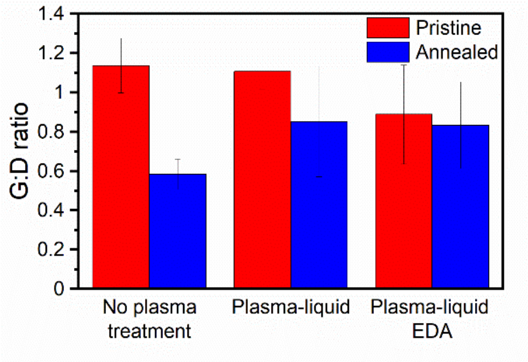

Figure S9. Summary of the g-band to d-band area ratios determined by Raman spectroscopy. The only clear change is that the pre-annealed samples have a typically lower G:D ratio, however this is recovered to a degree during the plasma processing.

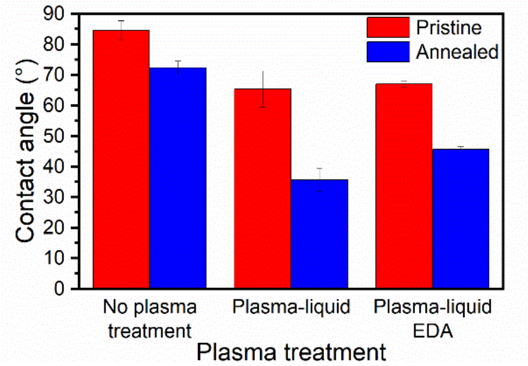

Figure S10. Summary of the contact angle measurement results for the 6 different carbon nanotube treatments. All treatments including simple annealing reduce the contact angle, though combining annealing with plasma-liquid yields the most significant modification.

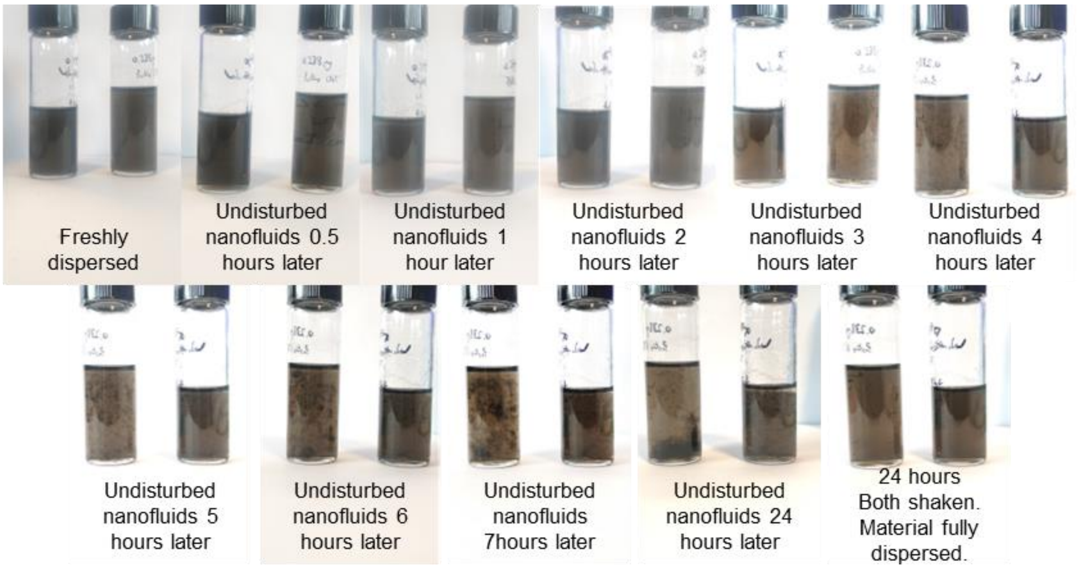

Figure S11. Photographs highlighting the short-term flocculation effect on stored nanofluids without disturbing. The images have been colour-corrected to emphasise the flocculation of material.

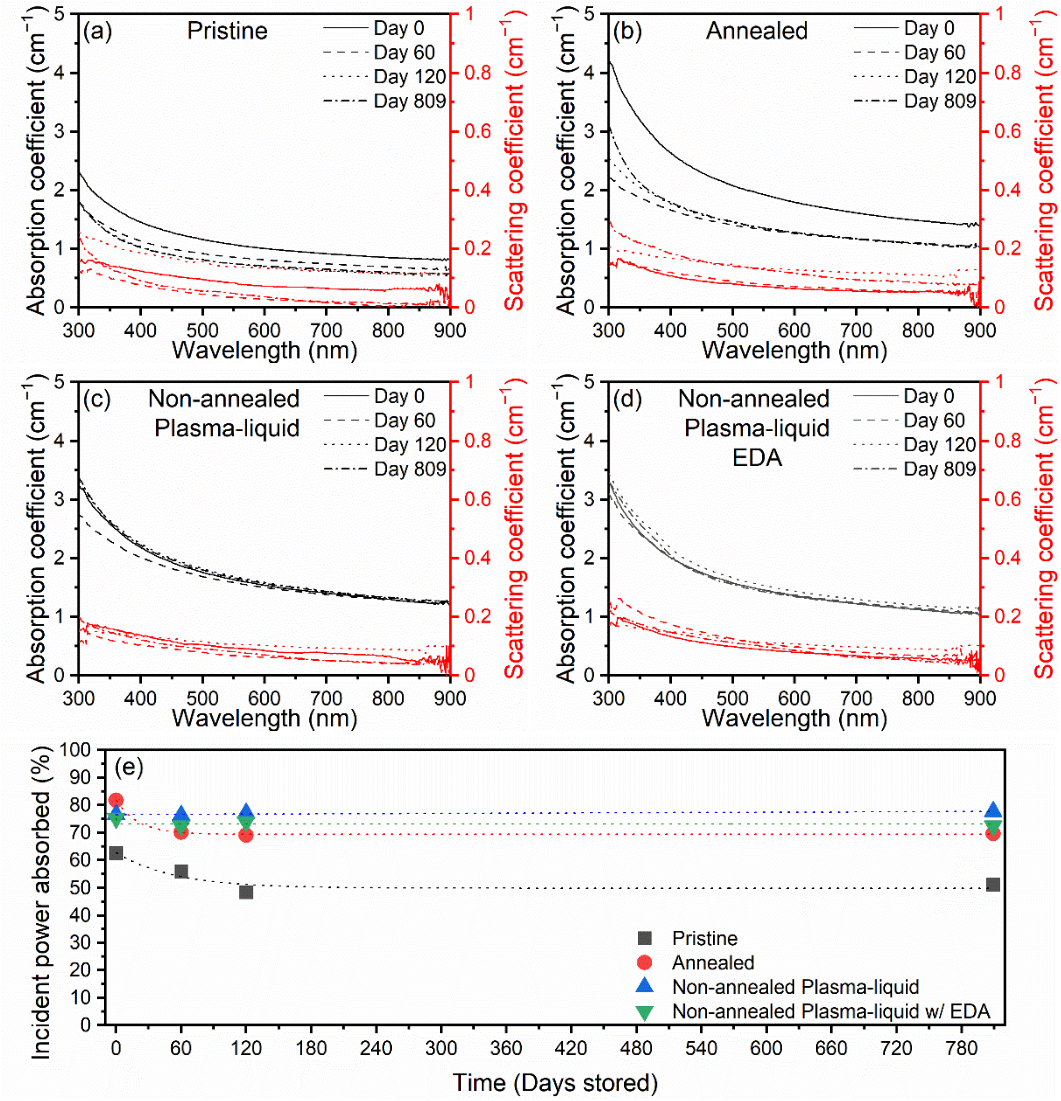

Figure S12. The values of absorption and scattering coefficients obtained from ultraviolet-visible spectroscopy via the transmittance port and an integrating sphere (**a**–**d**). The absorption coefficients are seen to stay relatively stable for the plasma-treated samples, however the annealed sample shows a diminishment with storage time. The percentage of incident power absorbed for each carbon nanotube-based nanofluid over 120 days is presented in the fourth chart (**e**).

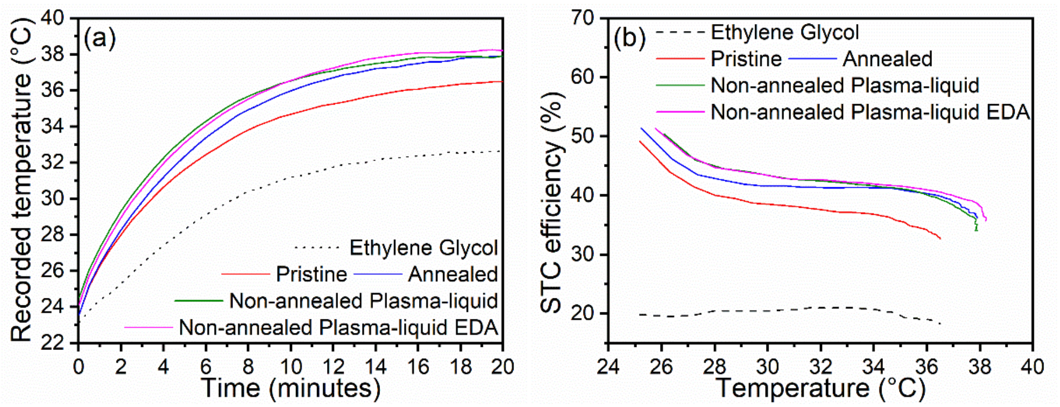

Figure S13. (**a**) Variation in temperature of the nanofluids and ethylene glycol over 20 min of exposure to simulated solar radiation, with small improvements noted for the plasma-

treated and annealed samples. (**b**) The resultant solar thermal conversion efficiency after accounting for heat lost.

S5. Further images on sample stability

Figure S14 illustrates the stability enhancement offered by the "plasma–liquid" treatment over the "pristine" material. Even in a stagnant fluid the dispersion is visible improved over the "pristine" sample. This sedimentation is anticipated to be prevented in real-world applications where circulation and shear thinning can ensure continuous mixing.

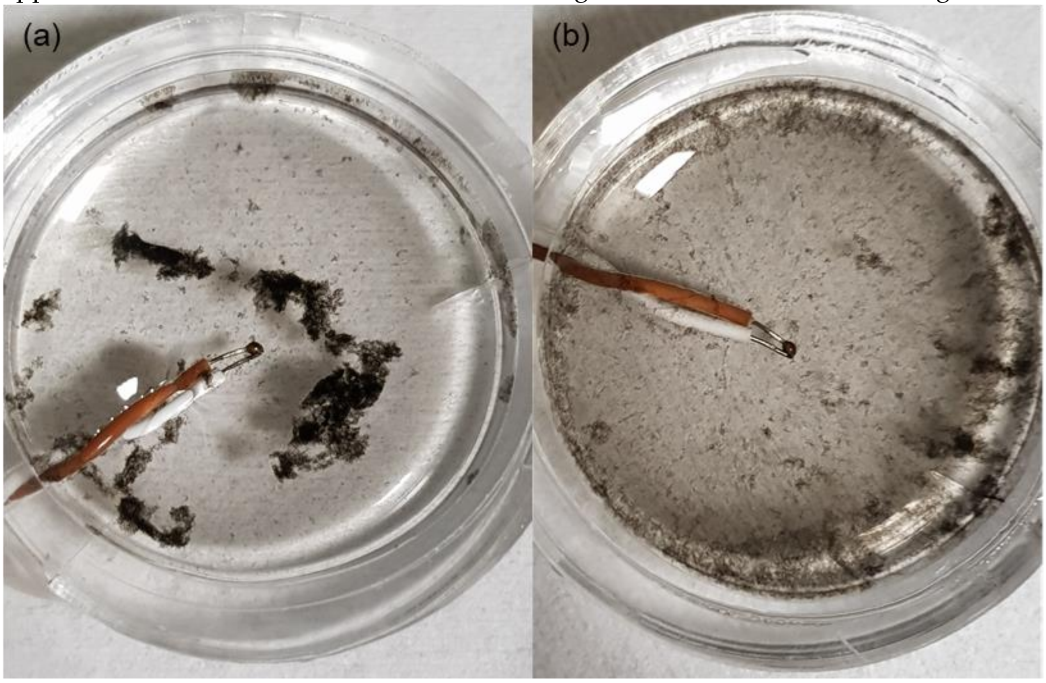

Figure S14. Photographs taken during the solar-thermal conversion experiments, (**a**) shows the "pristine" sample whereas (**b**) is a photograph of the "plasma–liquid" conditions.

References

1. *ASTM G173-03*; ASTM Standard Tables for Reference Solar Spectral Irradiances: Direct Normal and Hemispherical on 37° Tilted Surface 2012. ASTM International: West Conshohocken, PA, USA, 2020.
2. Pak, B.C.; Cho, Y.I. HYDRODYNAMIC AND HEAT TRANSFER STUDY OF DISPERSED FLUIDS WITH SUBMICRON METALLIC OXIDE PARTICLES. *Exp. Heat Transf.* **1998**, *11*, 151–170. https://doi.org/10.1080/08916159808946559.
3. Zhou, S.-Q.; Ni, R. Measurement of the Specific Heat Capacity of Water-Based Al2O3 Nanofluid. *Appl. Phys. Lett.* **2008**, *92*, 093123. https://doi.org/10.1063/1.2890431.
4. Incropera, F.P.; Dewitt, D.P.; Bergman, T.L.; Lavine, A.S. *Fundamentals of Heat and Mass Transfer*, Wiley: Hoboken, NJ, USA, 2007.
5. Chen, M.; He, Y.; Zhu, J.; Wen, D. Investigating the Collector Efficiency of Silver Nanofluids Based Direct Absorption Solar Collectors. *Appl. Energy* **2016**, *181*, 65–74. https://doi.org/10.1016/j.apenergy.2016.08.054.
6. Delfani, S.; Karami, M.; Akhavan-Behabadi, M.A. Performance Characteristics of a Residential-Type Direct Absorption Solar Collector Using MWCNT Nanofluid. *Renew. Energy* **2016**, *87*, 754–764. https://doi.org/10.1016/j.renene.2015.11.004.
7. Gorji, T.B.; Ranjbar, A.A. A Numerical and Experimental Investigation on the Performance of a Low-Flux Direct Absorption Solar Collector (DASC) Using Graphite, Magnetite and Silver Nanofluids. *Sol. Energy* **2016**, *135*, 493–505.

https://doi.org/10.1016/j.solener.2016.06.023.
8. Jeon, J.; Park, S.; Lee, B.J. Analysis on the Performance of a Flat-Plate Volumetric Solar Collector Using Blended Plasmonic Nanofluid. *Sol. Energy* **2016**, *132*, 247–256. https://doi.org/10.1016/j.solener.2016.03.022.
9. Moncrieff, D, A.; Barker, P, R. Secondary Electron Emission in the Scanning Electron Microscope. *Scanning* **1978**, *1*, 195–197.
10. Inada, H.; Su, D.; Egerton, R.F.; Konno, M.; Wu, L.; Ciston, J.; Wall, J.; Zhu, Y. Atomic Imaging Using Secondary Electrons in a Scanning Transmission Electron Microscope: Experimental Observations and Possible Mechanisms. *Ultramicroscopy* **2011**, *111*, 865–876. https://doi.org/10.1016/j.ultramic.2010.10.002.
